# Cancer patients’ participation in population-based health surveys: findings from the HUNT studies

**DOI:** 10.1186/s13104-015-1635-5

**Published:** 2015-11-05

**Authors:** Sophie D. Fosså, Alv A. Dahl, Arnulf Langhammer, Harald Weedon-Fekjær

**Affiliations:** National Advisory Unit for Late Effects after Cancer Therapy, Oslo University Hospital, Radium Hospitalet and Cancer Registry of Norway, P.O.Box 4953, Nydalen, 0424 Oslo, Norway; Faculty of medicine, University of Oslo, Oslo, Norway; HUNT Research Centre, Department of Public Health and General Practice, Norwegian University of Science and Technology, Levanger, Norway; Oslo Center for Biostatistics and Epidemiology, Research Support Services, Oslo University Hospital, Oslo, Norway

**Keywords:** Population-based surveys, HUNT studies, Participation rates, Cancer patients, Sex, Age

## Abstract

**Background:**

The magnitude of participation bias due to non-participation should be considered for cancer patients invited to population-based surveys. We studied participation rates among persons with and without cancer in a large population based study, the Nord-Trøndelag Health Study (HUNT).

**Methods:**

Citizens 20 years or above living in the Nord-Trøndelag County of Norway have been invited three times to comprehensive health surveys. The invitation files with data on sex, invitation date and participation were linked to the Cancer Registry of Norway. In a first step unadjusted crude participation rates (participants/invited persons) were estimated for cancer patients (CaPts) and non-cancer persons (NonCaPers), followed by logistic regression analyses with adjustment for age and sex. To evaluate the “practical” significance of the estimated odds ratios in the cancer diagnosis group, relative risks were also estimated comparing the observed rates to the estimated rates under the counterfactual assumption of no earlier cancer diagnosis among CaPts.

**Results:**

Overall 3 % of the participants in the three HUNT studies were CaPts and 59 % of them had been diagnosed with their first life-time cancer >5 years prior to each survey. In each of the three HUNT surveys crude participation rates were similar for CaPts and NonCaPers. Adjusted for sex and age, CaPts’ likelihood to participate in HUNT1 (1984–86) and HUNT2 (1995–97), but not in HUNT3 (2006–2008), was statistically significantly reduced compared to NonCaPers, equaling a relative risk of 0.98 and 0.96, respectively. The lowest odds ratio emerged for CaPts diagnosed during the last 2 years preceding a HUNT invitation. Only one-third of CaPts participating in a survey also participated in the subsequent survey compared to approximately two-thirds of NonCaPers, and 11 % of CaPts participated in all three HUNT surveys compared to 37 % of NonCaPers.

**Conclusion:**

In the three HUNT surveys no or only minor participation bias exist as to CaPts’ participation rates. In longitudinal studies selection bias as to long-term cancer survivorship should be taken into account, the percentage of repeatedly participating CaPts diminishing more strongly than among NonCaPers.

## Background

Many countries in the Western world have public registries which cover the entire population or specified sub-groups reflecting the prevalence and incidence of major adverse health conditions like cardiovascular diseases or cancer. Population-based surveys based on questionnaires have the potential to provide additional knowledge about self-reported physical and psycho-social health in large cohorts including information on overall wellbeing and life style. Linked together with data from public registries findings from population-based cross-sectional or longitudinal surveys provide extended data about the health in the general population. Such data may be used when national health-related interventions are designed.

Among other factors the validity of data from population-based surveys depends on the participation rate among the invited individuals. Non-participation increases the risk of selection bias which easily occurs in surveys that are not based on randomly selected individuals.

According to the medical literature, non-participation in general health surveys is associated with low socio-economic status (low education, low income, no health insurance), reduced general health, and increased post-survey mortality of the invited individuals [1–9]. The participation rates in studies of persons with specific diseases (diabetes mellitus, mental disorders, chronic lung diseases, cardiovascular diseases, diabetes mellitus) have been analyzed in several reports [5, 6, 8–10] whereas comparable rates among person with malignant diseases have been published less often. When analyzed, the participation rates of persons with cancer seemed to be relatively low [11, 12].

The Nord-Trøndelag Health Studies (Helseundersøkelse i Nord-Trøndelag, HUNT) have collected individual population-based data through three surveys: HUNT1 (1984–86), HUNT2 (1995–97), and HUNT3 (2006–08) [13]. Besides recording of basic socio-demographic data (age, education, civil status), these surveys have covered common adverse health conditions. The HUNT surveys are considered to be representative of the health situation of the total Norwegian population. Results of the HUNT surveys have so far resulted in approximately 600 peer-reviewed papers in national and international journals.

Data from the HUNT studies enable studies of the health of cancer patients (CaPts) to matched controls without cancer (NonCaPers), all identified among the HUNT participants (internal comparisons). Data from NonCaPers HUNT-participants have been used as controls when clinical cancer samples are studied (external comparisons) [14–16]. However, the participation rates of CaPts compared to NonCaPers, factors associated with participation and eventual inter-survey differences have not been focus of any report so far.

Based on a questionnaire sent to non-participants 9 months after HUNT3 the percentages of persons with a cancer diagnosis did not differ between participants and non-participants, with only marginal differences between males and females. [9] This result might suggest absence of any major selection bias related to participation rates among CaPts. However, this study was based on non-participants’ self-report and did not characterize the CaPts as to age, cancer type or time since diagnosis. Based on the unique person number given to all inhabitants who live in Norway, there is an opportunity to link all persons invited to the HUNT surveys to the Cancer Registry of Norway (CRN). Accordingly, all invited CaPts can be identified enabling further characterization of the participating and non-participating CaPts on demographic and selected medical variables. These observations an then be compared to corresponding results emerging among NonCaPers.

On this background the overall aim of our study was to describe eventual selection bias and its magnitude in the HUNT surveys related to CaPts’ participation. Their participation rates were compared to those observed among NonCaPers using a cross-sectional design for each of the three surveys, followed by a longitudinal analysis which addressed the individuals’ participation in subsequent surveys.

## Methods

### Sample recruitment

The current analyses were based on individualized record linkage between the three HUNT surveys and the CRN based on the unique Norwegian person number used by all public and large clinical registries.

#### The HUNT surveys

All inhabitants aged 20 years and above living in the county of Nord-Trøndelag of Norway have been invited to participate in three consecutive health surveys [13]. In all surveys data were collected by questionnaires and clinical examinations addressing several adverse health issues [cardiovascular disease, respiratory diseases, cancer, mental distress (anxiety, depression), and musculo-skeletal diseases]. The aims of each survey were described in an invitation letter sent to all eligible persons together with the first questionnaire (Q1). All invited persons were encouraged to attend a subsequent standard examination of height, weight, blood pressure, and blood sampling taking part in their local municipality. Q1 was returned at this examination, and the persons were asked to complete additional questionnaires. The present study is based on data from the returned Q1 questionnaire of each survey.

### The Cancer Registry of Norway

The CRN was established in 1951, and since 1953 clinicians and pathologists in Norway have been obliged by law to submit individualized information about all new cancer diagnoses to the CRN. For each patients the CRN collects information on demographic variables (sex, age, residence), cancer type, date of diagnosis, initial treatment and date and cause of death. The CRN covers 99 % of all new cancer diagnoses in persons living in Norway [17]. Based on data from the CRN the date of the first-time malignancy-proving biopsy determines the start of a patient’s cancer trajectory. However, 3–8 weeks usually elapse before the patients are informed about their malignancies, treatment and prognosis categorizing them as persons expected to be aware of their malignant diagnosis.

 For each person invited to one of the HUNT surveys cancer-related information was retrieved from the CRN, excluding all records on non-melanoma skin cancer. Due to data protection the date of a cancer diagnosis as well as the date of death was set to the 15th within the true month and year. In cancer patients with multiple malignancies only the first diagnosis was considered.

### Definitions and data management

Participants were defined as persons returning the Q1 questionnaire of each particular HUNT survey. The crude participation rates depict the number of Q1-participants related to the number of invited persons calculated separately for NonCaPers and CaPts. Due to the imprecise day in the date of cancer diagnosis and an eventual delayed information to a patient about his/her most recent malignant diagnosis, CaPts were regarded as NonCaPers if their first-time cancer diagnosis preceded the survey invitation date by 2 months or less. The time from a cancer diagnosis to a HUNT invitation (“diagnostic interval”) was categorized as: <2 years, 2–5 years and >5 years. Participation rates were calculated within each of these time categories.

The files extracted from the HUNT surveys for the current project contained the dates of survey invitation, the persons’ age at invitation (categorized as <50, 50–59, 60–69, 70–80 and 80+ years), participation status (Yes/No), and information on date of death or emigration from the county. Individuals who had died or emigrated prior to the date of invitation were removed before further analysis.

### Statistics

Descriptive within-group and between-group comparisons were performed using non-parametric descriptive and analytic methods [median (range), Mann–Whitney U test, Chi square test].

With age and sex being independent variables logistic regression analyses were performed separately for each survey and the participation rates of NonCaPers being the reference. A challenge with the logistic regression analysis was the interpretation of odds ratios due to high proportions of participation. Generally, odds ratios are often interpreted as approximated relative risks, but this approximation is only valid for rare events. For frequent events such as participation in the HUNT surveys, estimated odds ratios can seem large in the absence of substantial differences. Hence, we also estimated a corresponding predicted relative risk given by the ratio between the observed attendance among CaPts and the estimated attendance for the same group under the counterfactual assumption of no earlier cancer diagnosis. This estimated relative risk can be seen as an estimate of the real life differences which could be expected from the estimated odds ratios among CaPts. An alternative calculation using all the data gave similar results.

Statistical significance was defined by p < 0.05. Analyses were performed using the SPSS version 18 and STATA version 13 software packages.

## Results

### Crude participation rates

The participation rates in HUNT1, HUNT2 and HUNT3 were 90, 70 and 55 %, respectively with 2, 3 and 5 % of the participants being CaPts. In HUNT1 and HUNT2 the participation rates were similar for CaPers and NonCaPers (Table 1). In HUNT3 the participation rate of CaPts exceeded that of NonCaPers (58 vs 54 %, p < 0.001).

**Table 1:** Number of participants and participation rates among persons without cancer (NonCaPers) and those with a cancer diagnosis (CaPts)

	HUNT1 [n 85,516 (90 %)]^a^				HUNT2 [n 92,406 (70 %)]^a^				HUNT3 [n 93,552 (55 %)]^a^			
	NonCaPers		CaPts		NonCaPers		CaPts		NonCaPers		CaPts	
Invited	83,465		2051		89,301		3105		89,188		4364	
Participating	75,083	(90 %)	1837	(90 %)	62,899	(70 %)	2171	(70 %)	48,159	(54 %)	2511	(58 %)
Sex^b^												
Males	36,915	(88 %)^c^	760	(89 %)	29,557	(66 %)	931	(70 %)	21,822	(49 %)	1167	(57 %)
Females	38,168	(92 %)	1077	(90 %)	33,342	(75 %)	1240	(69 %)	25,336	(59 %)	1344	(58 %)
Age (years)^b^												
Median (range)	47 (19–101)^d^		69 (22–99)^e^		48 (19–104)		70 (20–95)		53 (19–101)		69 (21–96)	
<70	63,782	(90 %)	1008	(91 %)	53,298	(71 %)	1120	(78 %)	41,216	(54 %)	1396	(64 %)
≥70	11,301	(89 %)	829	(87 %)	9601	(68 %)	1051	(63 %)	6943	(55 %)	1115	(51 %)
Years since cancer diagnosis^b^												
Median (range)			5.1 (0.2–33)				6.8 (0.2–44)				7.0 (0.2–54)	
<2			397	(86 %)			374	(67 %)			441	(46 %)
2–5			451	(91 %)			479	(68 %)			531	(58 %)
>5			989	(90 %)			1318	(71 %)			1539	(58 %)
Cancer type^b^												
Breast			392	(89 %)			394	(71 %)			501	(60 %)
Colorectal			245	(89 %)			306	(68 %)			367	(55 %)
Prostate			184	(88 %)			247	(66 %)			438	(64 %)
Lung			19	(86 %)			29	(59 %)			48	(49 %)
Melanoma			102	(90 %)			169	(80 %)			255	(61 %)

^a^Number of all invited persons per HUNT survey (participation rate)

^b^Participants only

^c^% participating individuals (among those invited in the indicated category)

^d^Non-participating NonCaPers: HUNT1: 35 years (20–102); HUNT2: 38 years (20–104); HUNT3: 42 years (20–103)

^e^Non-participating CaPts: HUNT1: 73 years (22–98); HUNT2: 77 years (20–100); HUNT3: 74 years (20–101)

In all three surveys the diagnostic interval exceeded 5 years for more than 50 % of the CaPts. (median 5.1, 6.8 and 7.0 years for HUNT1, 2 and 3, respectively). The lowest participation rates were observed among CaPts diagnosed within 2 years preceding the HUNT invitation (Table 1). This participation rate decreased from HUNT1 to HUNT2 and further to HUNT3. In HUNT3 only 46 % of the invited CaPts participated if their diagnosis was made during the last 2 years, opposed to 86 % in HUNT1. Type of malignancy had limited impact on CaPts’ participation. Among the five types of CaPts those with lung cancer, the malignancy with the poorest prognosis, had the lowest participation rate in each of the HUNT surveys.

### Sex

In each HUNT survey the participation rates among NonCaPers were lower in males compared to females, with 11 % difference in HUNT3 (Table 1). Much smaller sex differences were observed among participating CaPts, and in HUNT3 the participation rates among male CaPts even exceeded that of NonCaPers (57 vs. 49 %, p < 0.001).

### Age

Participating CaPts were generally 20 years older than NonCaPers (Table 1). Non-participating NonCaPers were 10–15 years younger than attending NonCaPers, whereas the comparable difference in CaPts ranged from 4 to 7 years. In HUNT2 and HUNT3, more CaPts aged less than 70 years than comparable NonCaPers participated [p < 0.001 for HUNT2 (78 vs. 71 %) and HUNT3 (64 vs. 54 %)], with opposite findings for individuals 70 years old or older (HUNT2: 63 vs 68 %; HUNT3: 51 vs 55 %).

### Age and sex

**Fig. 1:**
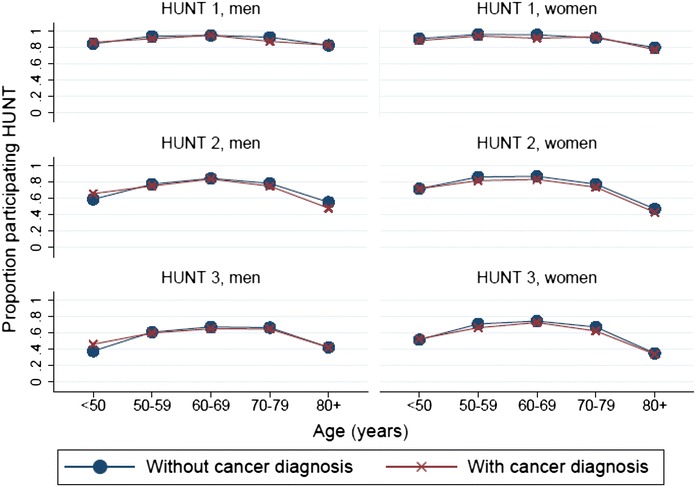
Participation rates for persons with or without a previous cancer diagnosis in HUNT1, HUNT2 and HUNT3, separately for men and women, according to five age categories

Within each of the sub-categories of age and separately for males and females the participation rates between CaPts and NonCaPers were small, none of them exceeding 10 % (Fig. 1). According to the multivariate logistic regression analyses (after adjustment for age and sex) CaPts were shown to be significantly less likely than NonCaPers to participate in HUNT1 and HUNT2, (HUNT1: by 20 %; HUNT2: by 13 %), with no such difference emerging for HUNT3. However, the practical differences were minor with estimated corresponding relative risks of 0.98 and 0.96 (Table 2). Further analyses proved that significantly reduced odds ratios were only observed among CaPts from HUNT1 and HUNT2 whose diagnostic interval was less than 2 years (reduction by 39 and 27 % in HUNT1 and HUNT2, respectively) (Table 3).

**Table 2:** Estimated odds ratios for crude participation rates for cancer patients in HUNT surveys: unadjusted and adjusted odds ratios for all persons with vs. without previous cancer diagnosis (reference), plus corresponding predicted relative risk (for easier interpretation of the adjusted odds ratios)*

	Odds ratio^*^			Predicted relative risk^**^
	No cancer (reference)	Cancer		Cancer
		Unadjusted	Adjusted for sex and age^***^	
HUNT1	1.0	0.96 {0.83, 1.11}	0.80 {0.68, 0.92}	0.98
HUNT2	1.0	0.98 {0.90, 1.05}	0.87 {0.80, 0.95}	0.96
HUNT3	1.0	1.15 {1.09, 1.23}	0.96 {0.90, 1.03}	0.98

^*^ With overall high participation rates, odd ratios must be interpreted with caution, and they are not good approximations of relative risks. Therefore use of predicted relative risks for easier interpretation

^**^ Relative risks given as the difference between the observed attendance in the cancer diagnosis group and the estimated attendance for the same group under the counterfactual assumption of no earlier cancer diagnosis (using multivariate logistic regression adjusting for sex and age). See “Methods” section for details

^***^ Age and age^2 (quadratic)

**Table 3:** Estimated odds ratios for crude participation rates for cancer patients in HUNT surveys: adjusted odds ratios for persons with cancer with varying time since diagnosis compared to persons without cancer diagnosis (reference)*

	Odds ratio^*^			
	No cancer (reference)	Cancer		
		Less than 2 years since cancer diagnosis	2–5 years since cancer diagnosis	5+ years since cancer diagnosis
HUNT1	1.0	0.61 {0.47, 0.81}	0.91 {0.67, 1.24}	0.85 {0.69, 1.05}
HUNT2	1.0	0.73 {0.61, 0.88}	0.87 {0.73, 1.03}	0.93 {0.83, 1.03}
HUNT3	1.0	0.94 {0.81, 1.09}	0.95 {0.83, 1.09}	0.97 {0.90, 1.05}

^*^ With overall high participation rates, odd ratios must be interpreted with caution and are not good approximations of relative risks

### Longitudinal assessment

Approximately one-third of the CaPts and two-thirds of NonCaPers participating in the first of two subsequent HUNT surveys also participated in the following HUNT survey (HUNT1 and HUNT2 or HUNT2 and HUNT3; Table 4). Eleven percent of the CaPts from HUNT1 participated in all three HUNT surveys, compared to about 37 % for individuals without cancer (p < 0.001).

**Table 4:**
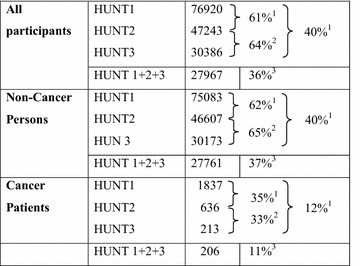
**Repeated participation in the three HUNT surveys**

^1^Percentage of HUNT1 participants attending HUNT 3

^2^Percentage of HUNT2 participants attending HUNT3

^3^Percentage of HUNT 1 participants with participation in all three surveys

## Discussion

After adjustment for age and sex CaPts’ likelihood to participate was significantly reduced in HUNT1 and HUNT2, but not in HUNT3. However, any differences in participation rates were small with the largest differences emerging in CaPts diagnosed within the last 2 years prior to a HUNT survey invitation. Respectively, one-third and approximately 10 % of the CaPts from HUNT1 participated in HUNT2 and HUNT3, the comparable figures more than tripled for NonCaPers.

During the last decades health survey participation rates have generally varied between 60 and 80 %, but have declined over time. [8] This trend was also demonstrated for all invited persons in the HUNT surveys [13], and confirmed for CaPts in the present study. Morton et al. [18] list some factors which may explain the variability of participation rates: differences in method of contact (mail, telephone), use of incentives, mandatory versus optional collection of biological material, and variability of the disease to be studied. The length and eventual sensitive nature of questionnaires may also be reasons why invited persons decline to participate [4], together with the opportunity of extensive data linkage. Full time work and rural residence have also been discussed as reasons for non-participation in general [11].

Analyses of multiple health surveys including the HUNT surveys have documented elevated non-participation rates for identifiable subgroups as socio-economically deprived individuals [1, 2, 6], and for those with reduced physical and mental health [3–6, 8, 9]. Associations with non-participation have been documented for cardiovascular diseases [9], chronic lung diseases [6, 19], but not for diabetes mellitus [10, 13]. The participation rates among males are often lower than among females [4, 19, 20] though Jacobsen et al. observed the opposite in the Rochester Epidemic Project [21]. In the HUNT surveys males, in particular younger men, had particularly low participation rates [13]. However, among CaPts participating in the HUNT studies the sex differences were much less, with more male than female CaPts participating in HUNT3.

The percentages of participating CaPts seem particularly low in reports if the patients are invited by cancer registries without involvement of the institution or the medical team responsible for the patients’ treatment. In Geller et al.’s study only 33 % of the invited cancer survivors participated [11], this percentage being in agreement with that observed by Jegou et al. [12]. These authors identified patients with colorectal cancer in a regional cancer registry and invited them to participated in future research. Only 37 % of all persons with rectal cancer were willing to be included in such a research-based cancer registry. This contrasts to the experience of Downing et al. [22] of a participation rate of 63 % among British persons with colorectal cancer. These authors included the names of the hospital director and of the chair of the patient’s responsible medical team in the head of the invitation letter. With this background, participation rates of 90 % (HUNT1), 70 % (HUNT2) and 58 % (HUNT3) among invited CaPts are surprisingly high. Among surveys specifically inviting CaPts [11, 12] non-awareness of the malignant diagnosis may be a reason for non-participation. In Nord et al’s [23] analysis of HUNT2, 26 % of non-participating CaPts identified by the CRN, claimed never to have been diagnosed with cancer.

Our findings support the suggestions by Langhammer et al. [9] of no or only minor differences between CaPts and NonCaPers as to participation in the HUNT studies, even though these authors’ observations were based on non-participants’ self-report by a questionnaire sent to them 9 months after the original invitation. In contrast, our study uses the CRN as basis of diagnostic validation. Therefore we are able to present more patient details than that study.

In agreement with Ness et al. [20] we observed the influence of the diagnostic interval on CaPts’ participation rate. In their US survey reduced “participation limitations” were reported among cancer patients whose cancer diagnosis preceded the survey for >5 years as compared to shorter intervals. In our study the reduced participation rates in CaPts with a diagnostic interval of <2 years may be explained by particularly poor health due to an aggressive malignancy or treatment-related side effects. Further, recently diagnosed cancer patients regularly have multiple contacts with the health care services during the first 2 post-diagnosis years, and many of them have not completed their initial treatment before 2 years have elapsed since diagnosis. They probably anticipate few benefits from participation in population-based HUNT surveys, and they therefore may be less motivated to participate. Researchers using the HUNT studies, and probably also other population-based health surveys, should therefore be aware of possible participation bias due to short diagnostic interval in cancer patients. Any statements comparing CaPers with NonCaPers may be less valid for cancer survivors less than 2 years after diagnosis.

Large differences in participation rates of CaPts and NonCaPers became more evident in the longitudinal assessment of participation. Approximately 60 % of the NonCaPers, but only one-third of the CaPts participated in two HUNT surveys, the comparable differences for attendance in all three surveys being respectively 37 and 11 %. The differences in repeated participation among persons with or without cancer leave comparisons of CaPts and NonCaPers problematic in longitudinal studies which aim to assess cancer patients’ health in subsequent surveys. On the other hand, the HUNT surveys allow valid analyses of health changes in long-term cancer survivors, if investigators are aware of the participation bias in the relatively few CaPts with repeated participation: Persons with a prior cancer diagnosis participating in more than one health survey are probably particularly healthy, the majority of them being without tumor activity of their first life-time cancer.

Our study has several limitations. First, the definition of cancer patients and thus the estimation of their participation rates can be debated since patients with a first life-time malignant diagnosis within the 2 months preceding the date of diagnosis as reported by the CRN were not included. The aim of our study was, however, to compare participation rates among NonCaPers with that of CaPts who were aware of their malignant diagnosis. Clinicians know that up to 2 months may elapse between performance of a diagnostic biopsy and patient information. In our view this justifies the exclusion of persons with a first life-time malignant diagnosis within 2 months from the cohort of CaPts, when participation rates were calculated. Retrospectively we could identify 185 persons who most probably were diagnosed with their first life-time cancer within 2 months prior to a HUNT invitation (HUNT1 47, HUNT2 61, HUNT3 77). Of these 105 individuals (57 %) did not participate in the relevant HUNT survey. From these figures we can stipulate that the non-participation rate for CaPts diagnosed within the two preceding would have been slightly higher, but without any principal change, if these patients had been reported as CaPts. Second, though we present results from three population-based studies the findings may not be valid for surveys in countries with different health care services. In countries without easy and non-expensive access to health care services, more persons will possibly participate in public health studies, than this was the case for the HUNT surveys. Third, we have only considered the first life-time cancer in CaPts whose records from the CRN could contain up to five malignant diagnoses. If the last date of cancer diagnosis had been taken into account, the participation rates within the diagnostic intervals of less than 5 years would have increased. Finally, adjustment for socio-economic status would have been desirable, but this information was not available during the performance of the analyses. The individualized data linkage with Norway’s populations-based registries is viewed as an essential advantage.

## Conclusion

We conclude that participation rates in each of the three HUNT surveys vary by sex and age, but that there are only minor differences between CaPts and NonCaPers. Differences are small and can probably be neglected in cross-sectional studies, though researchers should be aware of the particularly low participation of CaPts diagnosed less than 2 years prior to a HUNT invitation. In longitudinal analyses of the HUNT studies participation rates diminishes more rapidly among CaPts than among NonCaPts, leading to a risk of participation bias favoring surviving tumor-free CaPts.

## Abbreviations

HUNT: Nord-Trøndelag health study
CaPts: cancer patients
NonCaPers: persons without a cancer diagnosis
